# Effect of Nutrition Education Focusing on Dietary Quality on Cancer-Related Fatigue in Breast Cancer Patients: A 12-Week Randomized Controlled Trial

**DOI:** 10.3390/nu18060894

**Published:** 2026-03-12

**Authors:** Xinyi Miao, Jianyun He, Lan Cheng, Xinxin Cheng, Yuting Wang, Xiaoxia Lin, Zhenzhen Huang, Ran Wang, Shufang Xia

**Affiliations:** Wuxi School of Medicine, Jiangnan University, Wuxi 214122, China; miaoxinyi@stu.jiangnan.edu.cn (X.M.); hejianyun@stu.jiangnan.edu.cn (J.H.); chenglan@stu.jiangnan.edu.cn (L.C.); chengxinxin@stu.jiangnan.edu.cn (X.C.); wangyuting@stu.jiangnan.edu.cn (Y.W.); linxiaoxia@stu.jiangnan.edu.cn (X.L.); huangzhenzhen@stu.jiangnan.edu.cn (Z.H.); wangran@stu.jiangnan.edu.cn (R.W.)

**Keywords:** breast cancer, cancer-related fatigue, dietary quality, nutrition education

## Abstract

**Background**: Cancer-related fatigue (CRF) is a prevalent, persistent, and multidimensional symptom in breast cancer patients, negatively affecting physical function and quality of life (QoL). Dietary interventions have emerged as safe and cost-effective strategies to alleviate CRF. **Methods**: This assessor-blinded, randomized controlled trial evaluated the effects of a 12-week dietary quality-based nutrition education program on CRF in breast cancer patients. A total of 128 participants were randomly assigned to the intervention, which received nutrition education based on the Chinese Healthy Eating Index (CHEI), or the control group, which received standard care. Outcomes, including CRF (Revised Piper Fatigue Scale), dietary quality (CHEI), body mass index (BMI), self-management efficacy (Strategies Used by People to Promote Health, SUPPH) and QoL (Functional Assessment of Cancer Therapy-Breast, FACT-B) were assessed at baseline and post-intervention. **Results**: Of the 128 participants, 111 (86.7%) completed follow-up. Linear mixed-effects models demonstrated a significant group × time interaction for total RPFS scores. After adjusting for age, BMI, cancer stage, pain, anxiety, and depression, the intervention group showed a significantly larger reduction in RPFS scores (β = −1.426, 95% CI: −1.959~−0.893, *p* < 0.001, Cohen’s d = −0.97). In addition, after adjusting for the same covariates, significant improvements were observed in CHEI (β = 4.799, 95% CI: 1.383~8.215, *p* = 0.006, Cohen’s d = 0.75), SUPPH scores (β = 16.657, 95% CI: 12.557~20.758, *p* < 0.001, Cohen’s d = 1.65) and FACT-B scores (β = 12.688, 95% CI: 9.250~16.125, *p* < 0.001, Cohen’s d = 1.16) in the intervention group, all showing meaningful changes compared with the control group. **Conclusions**: Dietary quality-based nutrition education significantly alleviated CRF and improved other health-related outcomes in breast cancer patients, suggesting that nutrition education may be an effective strategy for managing CRF and supporting recovery during breast cancer treatment.

## 1. Introduction

Breast cancer is the second most common cancer worldwide and the leading cancer type among women. According to the 2022 Global Cancer Statistics report, approximately 2.3 million new cases of breast cancer were diagnosed globally, with an estimated 666,000 deaths [1]. Following breast cancer diagnosis and treatment, patients often experience a wide range of physical and psychological symptoms, such as fatigue, depression, pain, and sleep disturbances, all of which significantly impair quality of life (QoL) [2]. Among these symptoms, cancer-related fatigue (CRF) is one of the most prevalent, persistent, and debilitating symptoms reported by breast cancer patients throughout the treatment continuum. CRF is defined as a distressing, persistent, and subjective sense of physical, emotional, and/or cognitive tiredness or exhaustion related to cancer or its treatment that is disproportionate to recent activity and interferes with usual functioning [3]. The prevalence of CRF among cancer patients ranges from 11% to 99%, with an average prevalence of 49.7% in breast cancer patients [4]. Notably, CRF is particularly common during chemotherapy, with prevalence estimates ranging from 58% to 94% [5]. CRF is typically long-lasting, multidimensional, and poorly relieved by rest, with approximately one-third of breast cancer patients experiencing persistent symptoms 5 to 10 years post-treatment [6]. As a result, CRF significantly compromises physical functioning, psychological well-being, and overall QoL [7].

CRF is influenced by a variety of factors, including sociodemographic characteristics, tumor and treatment-related variables, psychological factors, and lifestyle behaviors [8]. Although only a subset of these factors are modifiable, they represent important and feasible targets for intervention. Accumulating evidence suggests that cancer patients frequently experience substantial changes in dietary behaviors following diagnosis and treatment, which may contribute to the development or exacerbation of CRF [9]. Dietary interventions, as safe, cost-effective, and easily modifiable lifestyle strategies, have therefore emerged as promising approaches for mitigating CRF [10]. Dietary behaviors are strongly shaped by patients’ knowledge, self-efficacy, and self-management during cancer treatment and may influence CRF through both direct and indirect pathways, including alterations in energy metabolism, immune function, and inflammation [11,12]. Recent research on dietary determinants of CRF has shifted from focusing on individual nutrients toward broader dietary patterns and overall dietary quality, enabling a more comprehensive assessment of habitual dietary intake and its health effects. Dietary quality reflects the overall adequacy, balance and health-promoting components derived from habitual diets and encompasses food diversity, nutritional balance, degree of food processing, and dietary behaviors [13,14]. As a comprehensive indicator of dietary intake, dietary quality has been widely applied in nutritional epidemiology to evaluate dietary behaviors and intervention effectiveness [15], and has been shown to predict major health outcomes, such as all-cause mortality, cardiovascular disease, and cancer risk [16]. The Chinese Healthy Eating Index (CHEI) is a validated and reliable tool for evaluating the dietary quality in the Chinese population [17], and has been extensively applied in diverse clinical populations, including patients with cancer [18], diabetes [19], frailty [20]. Our previous cross-sectional study showed that lower CHEI scores were negatively associated with CRF [21]. Consistently, a prospective cohort study reported that higher dietary quality, assessed using the Healthy Eating Index-2010, was significantly associated with lower overall fatigue and fatigue subscale scores [22]. Collectively, these findings suggest that dietary quality represents a modifiable and clinically meaningful factor in the management of CRF among breast cancer patients, underscoring the need for targeted strategies to improve overall dietary patterns.

From a theoretical perspective, the Integrated Theory of Health Behavior Change (ITHBC) provides a robust framework for understanding how nutrition education may influence dietary behaviors and subsequently alleviate CRF. The ITHBC posits that sustained health behavior change arises from the dynamic integration of condition-specific knowledge and beliefs, self-efficacy, and self-management skills, supported by ongoing professional guidance [23]. Within this framework, nutrition education serves as a key intervention strategy by enhancing patients’ dietary knowledge, strengthening dietary self-efficacy, and promoting sustained self-management of eating behaviors. These behavioral changes may lead to improvements in dietary quality and nutritional status and, in turn, contribute to the alleviation of CRF through improved energy balance and reduced inflammatory burden.

Despite growing evidence linking dietary quality to CRF, high-quality RCTs evaluating theory-driven nutrition education interventions targeting dietary quality in breast cancer patients remain limited. Guided by the ITHBC, we developed a theory-informed nutrition education program aimed at improving dietary quality among breast cancer patients experiencing CRF. The primary objective of this study was to evaluate the effects of a 12-week nutrition education intervention on CRF. Secondary objectives included assessing changes in dietary quality, nutritional status, self-efficacy, and overall QoL. We hypothesized that, compared with standard care, participants receiving the nutrition education intervention would demonstrate significantly reduced CRF, improved dietary quality, enhanced nutritional status, greater self-efficacy, and improved QoL.

## 2. Materials and Methods

### 2.1. Design Overview

This study was designed as a 12-week, prospective, two-arm, assessor-blinded randomized controlled trial (RCT). The trial was approved by the Medical Ethics Committee of the Affiliated Hospital of Jiangnan University (Approval No. LS2024542) and prospectively registered in the Chinese Clinical Trial Registry (ChiCTR2500096760). The study protocol complied with the Declaration of Helsinki, and the reporting of this trial followed the Consolidated Standards of Reporting Trials (CONSORT) guidelines.

The study was theoretically guided by the ITHBC. Based on key components of the theory—knowledge and beliefs, self-regulation skills, and social support—a structured nutrition education intervention was developed to promote the adoption and maintenance of health management behaviors. Individualized assessments, including dietary intake, CRF, nutritional status and other relevant health indicators, were conducted to inform a phased intervention program tailored to breast cancer patients with CRF undergoing chemotherapy. The intervention incorporated disease- and nutrition-related education, dynamic assessment of dietary quality with goal setting, and social support from family members and peers to enhance adherence and sustainability of behavior change.

### 2.2. Participants

Participants were recruited from the Affiliated Hospital of Jiangnan University between September and December 2024. The inclusion criteria were: (1) female patients with non-metastatic breast cancer; (2) diagnosis of CRF according to ICD-10 criteria; (3) aged ≥ 18 years; (4) postoperative patients receiving chemotherapy, with at least 3 months remaining before completion of the final chemotherapy cycle; (5) fully conscious and able to communicate; (6) possession of a smartphone for messaging and communication purposes; and (7) voluntary participation and provision of informed consent. The exclusion criteria were: (1) history of psychiatric disorders; (2) presence of other malignancies; (3) pregnancy or lactation; (4) comorbid conditions such as neurological or gastrointestinal diseases; (5) participation in any other interventional studies; (6) medical necessity for discontinuation; and (7) withdrawal of informed consent.

### 2.3. Sample Size Calculation

The sample size was calculated using a standard formula:

n = (Z_α_ + Z_β_)^2^ × 2σ^2^/δ^2^.

The sample size calculation was informed by a preliminary trial conducted by our research team (n = 12 per group). Such sample sizes are generally considered sufficient in pilot studies to provide estimates of means and standard deviations for formal sample size calculations, though the precision of these estimates can vary depending on the population and context [24]. In this trial, δ (1.08) is the expected mean difference in Piper Fatigue Scale scores between the intervention and control groups, and σ (1.43) is the pooled standard deviation. Assuming a two-tailed significance level (α) of 0.05 and a statistical power of 80%, the required sample size was calculated to be 28 participants per group. Sample size calculations were performed using G*Power software (version 3.1.9.7). Based on the preliminary trial data, the standardized effect size (Cohen’s d) was estimated at 0.76, indicating a large effect. Considering that preliminary trials may overestimate the true intervention effect due to sampling variability, and that CRF may vary substantially among breast cancer patients undergoing chemotherapy, we adopted a more conservative moderate effect size of Cohen’s d = 0.60 for the formal sample size calculation. This conservative estimate, which was pre-specified during the study planning stage, yields a required sample size of 88 participants (44 per group). To account for a 20% attrition rate, the total planned sample size was increased to 110 participants (55 per group). Considering the clinical heterogeneity of the breast cancer population and the need to enhance the robustness of intervention effect estimates, the study protocol allowed for adjustments to the sample size during recruitment, aiming at improving the statistical power and reducing the potential risk of Type II error, rather than for exploratory or subgroup analyses [25,26].

### 2.4. Randomization and Masking

The randomization sequence was generated by an independent researcher with no further involvement in the trial using the Research Randomizer website (https://www.randomizer.org/, accessed on 1 September 2024). The sequence was created prior to participant enrollment and implemented through sequentially numbered, opaque, sealed envelopes to ensure adequate allocation concealment. After written informed consent had been obtained, baseline assessments were conducted by independent assessors who were blinded to the group allocation. These assessors were responsible only for data collection before and after the intervention and did not participate in the implementation of the intervention. Participants were subsequently randomized in the order of enrollment by sequentially opening the envelopes, resulting in a 1:1 allocation to the intervention or control group. Due to the nature of the nutritional intervention, blinding of participants and intervention providers was not feasible. However, group allocation was also concealed from data analysts throughout the study to minimize the risk of analytical bias. After randomization, participants in the intervention group received a 12-week dietary-quality-based nutrition education program and were invited to join a WeChat group for online nutritional support. To minimize potential bias and contamination, several measures were implemented, including ward-level separation of participants, standardized training for outcome assessors, individualized interventions conducted in separate rooms, and restricted circulation of educational materials.

### 2.5. Intervention

Patients in the intervention group received four in-hospital education sessions based on dietary quality and 12 weeks of online nutritional support. The intervention consisted of four themes delivered during inpatient chemotherapy (face-to-face) and at home (online): (1) CRF and dietary quality management (Weeks 1–3); (2) Symptom management and dietary adjustment (Weeks 4–6); (3) Knowledge consolidation and dietary habit modification (Weeks 7–9); (4) Dietary self-assessment and self-care (Weeks 10–12). Each patient received the Dietary Guidebook for Breast Cancer Patients before discharge following the first face-to-face intervention to guide their dietary practices during the home-based phase. This brochure covered: (1) diet and disease; (2) dietary guidelines for breast cancer patients; (3) the Chinese Dietary Guidelines for Chinese Residents (2022); and (4) dietary recommendations for breast cancer patients undergoing chemotherapy. During the intervention, patients with lower dietary quality received individualized dietary guidance based on dietary quality scores, including face-to-face education, feedback on dietary records, and dietary adjustments related to chemotherapy symptoms, while patients with higher dietary quality were encouraged to maintain healthy eating habits. To ensure consistency of the intervention, a standardized dietary handbook, dietary quality scoring and assessment tools were developed, and an operational manual was developed for training interventionists, with adherence monitored through supervision and record checks. Within this standardized framework, individualized adjustments were allowed based on patients’ dietary quality, symptoms, and lifestyle, enabling a scientific and feasible dietary quality-based nutrition education. During the home-based intervention phase, participants received remote nutritional counseling via WeChat or telephone, including answers to diet-related questions and reminders to record dietary intake. Daily photographic records were collected to assess dietary quality, and brief feedback was provided according to the Chinese Dietary Guidelines for Chinese Residents and breast cancer-specific dietary recommendations to reinforce positive behaviors and offer suggestions for improvement. Nursing staff concurrently monitored adherence to the recommended dietary plan. The detailed intervention content and delivery procedure are shown in Table 1.

Patients in the control group received standard care. During hospitalization, in accordance with standardized nursing procedures and operational protocols of the breast surgery department, patients were provided with basic guidance related to the disease and its treatment, including information on breast cancer, medication-related instructions, and routine standard care during the postoperative and chemotherapy periods. In addition, healthcare professionals explained general principles for managing treatment-related adverse reactions and provided non-individualized dietary and nutritional advice, primarily limited to basic dietary principles and nutritional requirements. After discharge, routine follow-up was conducted via telephone or WeChat to address patients’ questions during the home-based phase.

Breast cancer patients scheduled to receive at least four cycles of adjuvant chemotherapy (21 days per cycle) were enrolled to minimize dropout rates, ensuring their participation throughout the 12-week intervention period. Adherence was assessed across four intervention stages. For the intervention group, engagement during each home-based phase was assessed using dietary report submission and diet-related interactions with the intervention providers. Participants were required to submit self-reported dietary records. Submission of two to three reports was assigned 2 points, one report 1 point, and no submission 0 points. Similarly, engagement with the intervention provider was evaluated according to the frequency of interaction regarding their diet: two or more interactions were scored as 2 points, one interaction as 1 point, and no interaction as 0 points. Each phase was scored on a scale of 0–4 points, resulting in a total adherence score ranging from 0 to 16 across all four phases. Adherence was categorized as high (13–16), moderate (8–12), low (1–7), and non-adherence (0) [27]. Participants with a score greater than 0 in any given phase were considered responsive to the intervention. If a participant received a score of 0 during any phase, the research team proactively contacted the individual to ascertain the reasons for non-participation. Those who could not be reached or declined to engage in online communication were classified as non-responsive. Identified barriers were addressed during subsequent face-to-face education sessions through targeted support or alternative strategies aimed at enhancing engagement. The response rate at each stage was calculated as the proportion of participants with high, moderate, and low adherence, excluding only non-adherent participants, out of the total number of participants initially allocated to the intervention group (n = 64). Participants from the control group consulted the intervention providers only when they had questions about the disease or treatment side effects, without completing structured tasks such as dietary reports or regular interaction sessions. As these consultations were sporadic and need-based, no formal quantitative adherence scoring system was established for the control group. To support participant engagement, telephone reminders were provided two days before each chemotherapy admission, and small incentives (e.g., water bottles, handheld massagers, or canvas bags) were offered after each intervention session for both groups.

### 2.6. Sociodemographic and Clinical Information

At baseline, participants’ sociodemographic and clinical characteristics were collected, along with lifestyle factors that could potentially influence CRF [8], including smoking status [28], alcohol consumption [29], and physical activity [30]. Physical activity was assessed using the International Physical Activity Questionnaire-Short Form (IPAQ-SF) and quantified as total weekly metabolic equivalents (MET-min/week). Participants were categorized into three activity levels: low (<600 MET-min/week), moderate (600 to 1500 MET-min/week), and high (>1500 MET-min/week). All data were collected by trained research staff and research assistants.

### 2.7. Primary Outcome

CRF was assessed using the Chinese version of the Revised Piper Fatigue Scale (RPFS), a 22-item tool designed to evaluate behavioral, affective, sensory, and cognitive dimensions of fatigue. The scale demonstrates strong internal consistency, with a Cronbach’s α of 0.91 [31]. Each item is scored on a 0 to 10 scale, with higher scores indicating the severity of fatigue. Both the dimension scores and the total score were calculated as the mean of the corresponding items. CRF severity was classified as mild (scores 1 to 3), moderate (scores 4 to 6), or severe (scores 7 to 10). CRF was reassessed after the end of the 12-week intervention.

### 2.8. Secondary Outcomes

#### 2.8.1. Dietary Intake Assessment and CHEI Calculation

Dietary intake was assessed using 24 h dietary recalls conducted over three nonconsecutive days [32]. The first recall was completed on the day of hospital admission, reflecting the patient’s dietary intake during the 24 h prior to admission. The following two recalls were performed post-discharge, once chemotherapy-induced gastrointestinal symptoms had subsided. Patients selected two days for the recalls, taking photographs of their food and sending them to the researchers via WeChat. The researchers and patients then verified the dietary intake through video or phone communication. To minimize dietary recall errors, two of the recalls were conducted on weekdays, and one was completed on a weekend day. The Nutrition Calculator v2.8.3.1 (Chinese Center for Disease Control and Prevention, Beijing, China) was used to estimate the average daily nutrient intake. The CHEI score was subsequently calculated following the procedures described in our previous publication [21]. The CHEI comprises 17 components: 12 adequacy components (e.g., whole grains, vegetables, fruits) and 5 moderation components (e.g., red meat, cooking oils). This index provides an evaluation of overall dietary balance and is independent of an individual’s economic status, making it applicable to a wide range of income groups [33].

#### 2.8.2. Nutritional Status Assessment

Nutritional status was assessed using body mass index (BMI) [34] and the Nutrition Risk Screening 2002 (NRS2002) [35]. All anthropometric measurements were performed in the hospital by trained professionals on the morning following hospital admission, with participants in a fasting state. Body weight was measured using a calibrated digital scale, and height was assessed with a non-stretchable anthropometric measuring tape, with participants standing barefoot against a wall, looking straight ahead, and keeping their shoulders relaxed. Measurements were obtained in the fasting state with participants wearing light clothing. Each parameter was measured three times at the same location to minimize measurement error, and the mean value was used for analysis. BMI was calculated as weight in kilograms divided by the square of height in meters (kg/m^2^). The NRS2002 includes three domains, disease severity, nutritional impairment, and age. A total score of ≥3 indicates a risk of malnutrition.

#### 2.8.3. Self-Management Efficacy

Self-management efficacy was assessed using the Strategies Used by People to Promote Health (SUPPH) scale [36], which measures patients’ confidence in performing health-promoting behaviors, managing symptoms, and coping with disease-related challenges. The Chinese version of the SUPPH has demonstrated strong reliability and validity, and it has been widely used in cancer populations [37]. The scale consists of 28 items, with higher scores indicating greater self-management efficacy.

#### 2.8.4. Quality of Life

QoL was assessed using the Functional Assessment of Cancer Therapy-Breast (FACT-B) scale [38], which includes breast cancer-specific items designed to capture concerns related to the disease and its treatment. The FACT-B comprises 36 items across five domains: physical well-being, social/family well-being, emotional well-being, functional well-being, and breast cancer-specific symptoms and issues. Total scores range from 0 to 144, with higher scores reflecting better QoL. The Chinese version of the FACT-B has been extensively validated and widely used in breast cancer populations, demonstrating robust reliability and validity [39].

### 2.9. Statistical Analysis

Statistical analyses were conducted using SPSS version 27.0 (IBM Corp, Chicago, IL, USA). The normality of continuous variables was assessed using the Shapiro–Wilk test. Normally distributed variables are presented as means ± standard deviations (SD), while non-normally distributed variables are reported as medians with interquartile ranges (IQR) [25th and 75th percentiles]. All analyses were conducted according to the intention-to-treat (ITT) principle, including all randomized participants. The primary outcome was the RPFS total and dimensional scores, while secondary outcomes (CHEI, BMI, NRS2002, SUPPH, and FACT-B total and subscale scores) were analyzed exploratorily. Linear mixed-effects models (LMMs) with random intercepts for participants were used to evaluate the effects of group (intervention vs. control), time (baseline vs. post-intervention), and the group × time interaction, which represented the primary intervention effect. Missing data were handled under the missing-at-random (MAR) assumption using LMMs. Three models were fitted: Model 1 (unadjusted); Model 2 (adjusted for age, BMI, and cancer stage); and Model 3 (further adjusted for pain, anxiety, and depression). Results are reported as β coefficients with 95% confidence intervals (CIs). All tests were two-tailed, with *p* < 0.05 considered statistically significant. Cohen’s d was calculated by dividing the group × time interaction coefficient (β) by the post-intervention residual standard deviation and interpreted as small (0.2), medium (0.5), or large (0.8) [40]. Supplementary conventional analyses were conducted using statistical tests: between-group comparisons with independent-samples *t*-tests or chi-square tests, and within-group changes with paired *t*-tests or Wilcoxon signed-rank tests, as appropriate. These supplementary analyses did not incorporate repeated measures, random effects, or covariate adjustments.

## 3. Results

### 3.1. Overview

**Figure 1:**
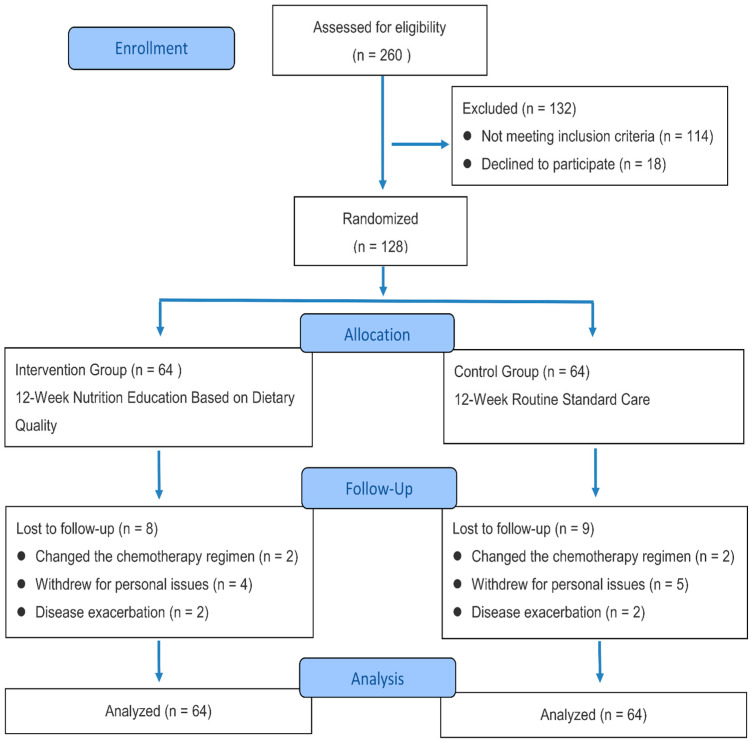
Participant recruitment flow during the trial in accordance with the CONSORT guidelines.

A total of 260 patients with breast cancer were screened for eligibility. Of these, 114 were ineligible and 18 declined participation. As a result, 128 CRF patients were ultimately enrolled and randomly assigned to either the intervention group (n = 64) or the control group (n = 64). The adjustment of sample size was methodologically driven and increased statistical power from the initially planned 80% to approximately 90%. During the study, 17 participants were lost to follow-up, with reasons including changes in chemotherapy regimens (n = 4), disease exacerbation (n = 4), and personal withdrawal (n = 9). In accordance with the ITT principle, the enrolled 128 participants were included in the final LMM analysis. The flow diagram of the study is shown in Figure 1.

### 3.2. Participant Baseline Characteristics

The baseline characteristics of the participants are summarized in Table 2. The median age of the participants was 56.50 years. The majority were post-menopausal (68.0%), diagnosed with stage II breast cancer (59.4%), and had undergone mastectomy (66.4%). No significant difference was observed in baseline characteristics, indicating successful randomization.

### 3.3. Patient Adherence and Response Rate

The detailed information on adherence data across the intervention period is shown in Table S1. High adherence was observed from 84.4% in Weeks 1–3 to 70.0% in Weeks 10–12. Moderate and low adherence levels slightly increased over time, while non-adherence remained low, ranging from 0% to 6.7%. The response rate decreased from 100% in Weeks 1–3 to 87.5% in Weeks 10–12, indicating gradual attrition during the study.

### 3.4. Effects on CRF

As shown in Table 3, for the total RPFS score, no significant main effects of group or time were observed in any model (all *p* > 0.05). However, the group × time interaction was consistently significant across Models 1–3 (all *p* < 0.001), indicating a greater reduction in CRF over time in the intervention group compared with the control group, independent of covariate adjustments. For Model 3, Cohen’s d was −0.97, indicating a large effect. In the subscales of RPFS (behavioral, affective, sensory, and cognitive fatigue), the main effects of group and time were generally non-significant after adjustment. However, a significant time effect for sensory fatigue in the unadjusted model was not retained in adjusted models. Notably, the group × time interaction remained statistically significant for all four dimensions in all models (all *p* ≤ 0.001), with Cohen’s d values in Model 3 ranging from −0.93 to −0.76, indicating moderate-to-large effects. Overall, sequential adjustment for age, BMI, cancer stage, pain, anxiety, and depression did not materially alter the results. The consistent and significant group × time interactions across both total and dimensional RPFS scores support a robust intervention effect on CRF.

### 3.5. Effects on CHEI Score

As shown in Table 4, regarding CHEI scores, no significant main effects of group or time were observed in any model (all *p* > 0.05). However, a significant group × time interaction was detected in the unadjusted model (Model 1: β = 4.780, 95% CI: 1.366~8.193, *p* = 0.006), and this effect remained statistically significant after adjusting for age, BMI, and cancer stage (Model 2: β = 4.820, 95% CI: 1.408~8.235, *p* = 0.006), as well as after further adjustment for pain, anxiety, and depression (Model 3: β = 4.799, 95% CI: 1.383~8.215, *p* = 0.006). In Model 3, Cohen’s d was 0.75, indicating a moderate effect. These findings indicate that the intervention was associated with a significantly greater improvement in CHEI scores over time in the intervention group compared with the control group, and this effect remained robust after sequential covariate adjustments.

### 3.6. Effects on BMI, Nutritional Status, and Self-Management Efficacy

As shown in Table 5, for BMI, no significant main effects of group or time were observed in any model (all *p* > 0.05). However, a significant group × time interaction was consistently identified across Models 1–3 (all *p* = 0.005), Cohen’s d in Model 3 was 0.10, indicating a minimal improvement in BMI over time in the intervention group compared with the control group. The results remained stable after adjustment for age, cancer stage, and additional clinical covariates (BMI was not included as a covariate in Model 2). For NRS 2002 scores, neither the main effects of group nor time, nor the group × time interaction, reached statistical significance in any model (all *p* > 0.05), suggesting no significant change between groups over time. For self-management efficacy (SUPPH score), no significant main effect of group was detected in any model (all *p* > 0.05). A significant time effect was observed in all models (all *p* < 0.001). Notably, the group × time interaction was highly significant in all models (all *p* < 0.001), with Cohen’s d values in Model 3 being 1.65, indicating a substantially greater improvement in self-management efficacy in the intervention group compared with the control group.

### 3.7. Effects on QoL

Table 6 summarizes the LMM analyses of the intervention on total and subscale scores of the FACT-B. For overall FACT-B scores, no significant main effect of group was observed (all *p* > 0.05), whereas a significant time effect was detected across all models (all *p* < 0.001). The group × time interaction remained consistently significant after full adjustment (all *p* < 0.001), with Cohen’s d values in Model 3 being 1.16, indicating greater improvement in overall QoL in the intervention group compared with the control group. Across subscales, no significant main effects of group were identified. Significant time effects were found for social/family well-being, functional well-being, and additional concerns (all *p* ≤ 0.01), as well as for physical well-being after adjustment (*p* ≤ 0.01), but no significant time effect was observed for emotional well-being. Significant group × time interactions were observed for social/family well-being (all *p* ≤ 0.05), emotional well-being (all *p* < 0.001), functional well-being (all *p* ≤ 0.05), and additional concerns (all *p* ≤ 0.01), while no significant interaction was found for physical well-being (all *p* > 0.05). In Model 3, Cohen’s d values for the significant group × time interactions ranged from 0.42 to 1.16, indicating moderate-to-large effects. Overall, the intervention significantly improved overall FACT-B scores and several of its domains, with results remaining robust after sequential covariate adjustment.

### 3.8. Supplementary Conventional Analyses

Supplementary conventional analyses using unadjusted statistical tests yielded results consistent with those from the LMM analysis, with no significant changes in the direction, magnitude, or statistical significance of the between-group differences in the primary outcome. Detailed results are provided in Tables S2–S5.

## 4. Discussion

Given the characteristics of CRF and the dietary habits of breast cancer patients undergoing chemotherapy, we implemented a dietary quality-based nutrition education program guided by the ITHBC theory. This 12-week RCT assessed the effects of the intervention on CRF and related health outcomes. Compared with standard care, patients who received the nutrition education demonstrated significant improvements in CRF, dietary quality, self-management efficacy and overall QoL. These findings collectively suggest that dietary quality-based nutrition education may serve as an effective supportive strategy to improve multiple dimensions of well-being in breast cancer patients with CRF.

Accumulating evidence suggests that diet plays an important role in both the development and management of CRF [10]. One RCT assigned breast cancer survivors with CRF to either a fatigue-reducing diet education group or a general health education control group, reporting greater improvements in CRF and sleep quality in the intervention group [41]. Importantly, the study controlled for potential psychological effects of educational contact by matching intervention frequency and duration between the groups. However, this intervention primarily focused on individual dietary components (e.g., fruits and vegetables), which limited the assessment of diet as an integrated pattern. Nowadays, dietary quality has emerged as a more critical determinant than individual nutrient intake [22]. A pilot RCT implementing an eight-week Mediterranean diet-based educational intervention reported significant reductions in CRF, particularly among participants (mostly breast cancer patients) with poorer baseline dietary quality [10]. Additionally, higher dietary quality and earlier, more regular meal timing were also reported to be associated with lower fatigue levels, suggesting that dietary interventions focusing on improving both dietary quality and meal regularity may contribute to alleviating CRF [42]. Our previous cross-sectional study demonstrated that the CHEI was associated with CRF among breast cancer patients, highlighting the importance of overall dietary patterns [21]. Building on these findings, the present 12-week RCT further demonstrates that a dietary quality-based nutrition education intervention can alleviate CRF in breast cancer patients undergoing chemotherapy. Notably, improvements were observed across behavioral, affective, sensory, and cognitive fatigue domains, suggesting that dietary interventions may exert multidimensional benefits in managing CRF. Therefore, the sustained improvements in dietary quality may represent a key pathway through which CRF symptoms were alleviated. Future studies should consider baseline dietary quality for inclusion or stratification while assessing dietary adherence, fatigue, and QoL, alongside relevant biological markers to elucidate potential mechanisms.

Nutritional education is widely recognized for its ability to improve patients’ nutritional knowledge and self-management abilities, thereby improving overall dietary quality [43]. In the present study, nutrition education, guided by the ITHBC framework and focusing on dietary quality, integrated disease-related education, personalized goal-setting and family engagement. This approach strengthened health beliefs and self-management capacity, resulting in significant improvements in CHEI scores among breast cancer patients. However, the effects of existing nutritional interventions targeting CRF are heterogeneous in terms of population characteristics, intervention duration, and delivery methods, which may partly explain the inconsistent findings across studies. For example, Shuremu et al. found that nutrition education based on Social Cognitive Theory substantially improved dietary diversity, with the intervention group being 7.7 times more likely to achieve diverse diets compared with the controls [44]. Similarly, a RCT demonstrated that personalized nutrition education delivered via a WeChat mini-program and telephone counseling improved both dietary intake and overall dietary quality among colorectal cancer survivors [45]. Given the variable trajectory of CRF in breast cancer, assessing overall dietary quality offers a more comprehensive understanding of dietary patterns and may lead to more targeted interventions. As this study evaluated only total CHEI scores, future research should further examine individual components to clarify their specific contributions to dietary quality and related health outcomes.

BMI is a key indicator of nutritional status [46]. In the present study, the group × time interaction was statistically significant across all three models, indicating that BMI increased slightly in the intervention group compared with the control group. These findings were different from the findings from another study, which showed that a shorter intervention period (6–8 weeks) did not result in improvements in BMI, suggesting that weight changes require a longer period for behavioral adaptation [47]. Since the CHEI is based on energy density rather than total energy intake, the higher consumption of healthy but energy-dense foods (e.g., nuts, legumes) may have contributed to a positive energy balance and increased BMI. This underscores the importance of monitoring total energy intake while improving dietary quality. Although nutritional risk during chemotherapy has been linked to CRF severity [48,49], the NRS-2002 scores in our study did not show significant changes, likely due to the short duration of the intervention and the participants’ high baseline BMI. Overall, dietary interventions typically require a longer duration to yield measurable effects on body weight and nutritional risk. By setting body weight as a long-term management target, our intervention aimed to enhance sustained dietary behavior changes, ultimately contributing to improved health outcomes.

Self-management efficacy reflects patients’ confidence in managing their disease, with higher levels facilitating the adoption and maintenance of healthy behaviors [50]. Health education can enhance risk perception and outcome expectations, thereby promoting positive health beliefs and behaviors [51]. In this study, a dietary quality-based nutrition education intervention, grounded in the ITHBC framework, significantly improved self-management efficacy in the intervention group compared with the control group. These findings are consistent with previous research, which reported that a 12-week multimodal dietary education program significantly enhanced dietary patterns, self-management efficacy, and social support among 430 middle-aged and older adults [52]. Furthermore, evidence also indicates that inadequate family support can hinder treatment adherence [53,54]. Integrating family and social support into interventions enables patients to better cope with disease, strengthen self-management efficacy, and promote the maintenance of long-term healthy behaviors. Therefore, providing structured knowledge, emotional support, and confidence-building strategies is critical for promoting self-management efficacy and facilitating persistent healthy behaviors.

CRF has detrimental effects on cognitive function, mood, and physical capacity, leading to substantial reductions in QoL and impairments in work, social engagement, and daily activities [3,55]. In this study, nutrition education based on dietary quality significantly improved overall QoL in breast cancer patients undergoing chemotherapy with concurrent CRF, which was consistent with the previous research that individualized dietary interventions, including nutrition education and targeted provision of macro- and micronutrients, have been shown to alleviate QoL-related symptoms in cancer patients [56]. Moreover, meta-analyses have shown a positive correlation between overall dietary quality and QoL among cancer survivors [57]. RCTs further demonstrate that adherence to healthy dietary patterns, such as the Mediterranean diet, or interventions that replace red and processed meats with alternative protein sources and refined grains with whole grains, can significantly enhance both overall and cancer-specific QoL [58,59]. Collectively, these findings underscore the potential of optimizing dietary patterns and providing individualized nutritional guidance as an adjunctive strategy to mitigate CRF and improve overall well-being in cancer patients.

Blinding of participants and intervention providers was not feasible due to the behavioral nature of the nutrition education intervention. Moreover, the intervention group received more structured and frequent contact with healthcare professionals than the control group, which may have introduced performance or attention bias. Increased contact with healthcare providers can enhance participants’ motivation, perceived support, and expectations of benefit [60], potentially influencing self-reported outcomes such as CRF, dietary quality, and QoL. Such influences are common in behavioral intervention research and should be considered when interpreting the findings. To minimize detection bias, outcome assessors and data analysts were blinded to group allocation, and validated instruments (e.g., RPFS and FACT-B) were used to ensure measurement reliability. Furthermore, the randomized controlled design and the consistent group × time interactions observed across multiple adjusted models strengthen the internal validity of the results. Future studies incorporating attention-matched control conditions and objective physiological measures would further improve methodological rigor and help isolate the specific effects of dietary education interventions.

Although this study employed an ITT approach including all randomized participants, the loss of 17 participants (approximately 13.3%) may have influenced the results. In ITT analyses, missing outcome data may attenuate between-group differences because incomplete observations can shift estimated means toward the overall average, potentially leading to conservative estimates of effect sizes [61]. If the responses of participants who withdrew differed systematically from those who completed the study, the observed Cohen’s d estimates may underestimate the true intervention effects. In this study, missing data were handled using LMMs under the MAR assumption, allowing all available data to be included and reducing potential bias associated with missing data [62]. Supplementary conventional analyses yielded similar trends, supporting the robustness of the main findings. Future studies should implement strategies to minimize dropout, such as increasing follow-up frequency, sending reminders via phone or email, providing flexible assessment schedules, or using online data collection [63]. Sensitivity analyses should also be pre-specified, for example, using multiple imputation or best/worst-case scenario analyses, to quantitatively assess the potential impact of missing data on study outcomes [62,64].

This study has several limitations. First, while a 3-day dietary recall was used, recall bias may still affect the accuracy of dietary intake reporting. Second, the lack of long-term follow-up prevents the evaluation of the sustainability of the intervention’s effects over time. Third, the sample size was modest and limited to a single tertiary hospital, which may restrict the generalizability of the findings to broader populations. Future research should implement blinded intervention designs and use objective measures, such as wearable monitoring devices, to more accurately assess participant behaviors. Long-term follow-up assessments at 6 and 12 months are recommended to evaluate the sustainability of the intervention effects over time. Multicenter studies including participants from diverse socioeconomic and cultural backgrounds are essential to determine whether the intervention is effective across different healthcare settings and populations. Such studies would not only allow for the evaluation of the intervention across different hospitals, communities, and patient groups but also help ensure that the findings are robust, broadly generalizable, and applicable to a variety of real-world settings. In addition, future studies could incorporate reinforcement strategies, such as periodic booster sessions, remote counseling, or digital reminders, to enhance adherence and maximize the long-term translational impact of the intervention.

## 5. Conclusions

In conclusion, a nutrition education program based on the ITHBC theoretical framework and focusing on dietary quality provides significant benefits for managing CRF in breast cancer patients undergoing chemotherapy. The intervention effectively reduced fatigue, improved dietary quality, and enhanced patients’ self-efficacy, leading to substantial improvements in overall QoL. These findings underscore the important role of individualized dietary guidance as part of a comprehensive approach to CRF management. By incorporating nutritional education into routine supportive care, healthcare providers can help improve both the immediate and long-term well-being of breast cancer patients, offering them a valuable tool for navigating the challenges of chemotherapy.

## Figures and Tables

**Table 1:** Intervention content and delivery process.

Intervention Theme	Intervention Timing and Frequency	Intervention Content	Online Support ^a^
CRF & Diet Quality (Weeks 1–3)	Second day of admission (Face-to-face education 30 min)	1. Introduce the concept of CRF, its prevalence, and potential adverse outcomes, enhancing patient awareness of CRF. 2. Distribute the “Breast Cancer Patient Dietary Guide,” provide personalized recommendations based on CHEI scores, educate patients and families, and set dietary goals together.	1. Online support was provided to address patients’ questions regarding disease, treatment, and dietary management. 2. Patients are reminded to submit dietary photos for three days. Individualized dietary adjustment plans are developed based on dynamic CHEI scores.
Symptom Management & Dietary Adjustment (Weeks 4–6)	First day of admission (Face-to-face education 30 min)	1. Review previous course content and re-educate patients on unfamiliar topics. 2. For patients with significant nausea or CRF, assess deficits in CHEI components and provide targeted improvements. Track changes in symptoms and diet over time to guide adjustments.	1. Collect dietary records and symptom diaries at each stage. 2. Report and provide feedback on CHEI trends at each stage to reinforce healthy dietary habits (e.g., reminders for hydration and balanced nutrition).
Knowledge Consolidation & Habit Optimization (Weeks 7–9)	Second day of admission (Face-to-face education 30 min)	1. Interactive Q&A sessions to clarify dietary misconceptions. 2. Educate patients and their families on scientific dietary and cooking practices, and provide recommended daily food intake to support accurate estimation of dietary needs.	1. Online review of patients’ dietary feedback records at each stage, providing guidance for patients to adopt appropriate dietary practices. 2. Encourage patients to share experiences in dietary and symptom management.
Dietary Self-Assessment & Self-Management (Weeks 10–12)	First day of admission (Face-to-face education 30 min)	1. Train patients to independently calculate the CHEI, with accuracy verified under researcher supervision, thereby enhancing patient autonomy in dietary management. 2. Provide individualized guidance for patients with low adherence, reinforcing healthy dietary habits; encourage the recording of dietary intake and symptoms to enhance self-efficacy. 3. Guide patients in setting long-term dietary goals based on dietary habits and CRF status.	1. Collect patient feedback and address issues related to diet and health management. 2. Regularly follow up on the patient’s health status and self-management progress to ensure the sustainability of behavior changes.

^a^ Weekly online support was delivered via WeChat or telephone. Abbreviations: CRF, cancer-related fatigue; CHEI, Chinese Healthy Eating Index.

**Table 2:** Comparison of baseline characteristics between the intervention and control groups.

Variable	Total (n = 128)	Intervention (n = 64)	Control (n = 64)	Z/χ^2^/t	*p*
Age (year) ^a^	56.50 (47.00, 63.00)	56.50 (48.25, 61.00)	56.00 (46.00, 66.00)	−0.415	0.678
BMI (kg/m^2^) ^d^	23.82 ± 3.26	23.87 ± 3.14	23.76 ± 3.39	0.262	0.793
Menopausal status ^b^					
Pre-menopausal	41 (32.0)	19 (29.7)	22 (34.4)	0.323	0.570
Post-menopausal	87 (68.0)	45 (70.3)	42 (65.6)		
Marital status ^c^					
Married	122 (95.3)	60 (93.8)	62 (96.9)	0.175	0.676
Widowed/divorced/single	6 (4.7)	4 (6.2)	2 (3.1)		
Education level ^a^					
Primary school or lower	33 (25.8)	15 (23.4)	18 (25.5)	−0.170	0.865
Middle school	32 (25.0)	19 (29.7)	13 (20.0)		
High school/secondary school	37 (28.9)	18 (28.1)	19 (29.0)		
Junior college or higher	26 (20.3)	12 (18.8)	14 (25.5)		
Employment ^b^					
Employed	29 (22.7)	12 (18.8)	17 (26.6)	1.230	0.541
Unemployed	29 (22.7)	16 (25.0)	13 (20.3)		
Retired	70 (54.6)	36 (56.3)	34 (53.1)		
Residence ^b^					
Rural areas	32 (25.0)	19 (29.7)	13 (20.3)	1.606	0.448
Towns	14 (10.9)	6 (9.4)	8 (12.5)		
Urban areas	82 (64.1)	39 (60.9)	43 (67.2)		
Family monthly income ^a^					
<3000 CNY	20 (15.6)	11 (17.2)	9 (14.1)	−0.784	0.433
3000~5000 CNY	64 (50.0)	33 (51.6)	31 (48.4)		
>5000 CNY	44 (34.4)	20 (31.2)	24 (37.5)		
Cancer stage ^a^					
I	37 (28.9)	19 (29.7)	18 (28.2)	−0.300	0.976
II	76 (59.4)	37 (57.8)	39 (60.9)		
III	15 (11.7)	8 (12.5)	7 (10.9)		
Surgery type ^b^					
Mastectomy	85 (66.4)	46 (71.9)	39 (60.9)	1.716	0.190
Lumpectomy	43 (33.6)	18 (28.1)	25 (39.1)		
Presence of comorbidities ^b^					
No	91 (71.1)	46 (71.9)	45 (70.3)	0.038	0.845
Yes	37 (28.9)	18 (28.1)	19 (29.7)		
VAS pain score ^a^	0.50 (0.00, 2.00)	1.00 (0.00, 2.00)	0.00 (0.00, 2.00)	−1.702	0.089
HADS-Anxiety score ^a^	5.00 (3.00, 8.00)	5.00 (3.00, 8.00)	5.00 (4.00, 8.00)	−0.656	0.512
HADS-Depression score ^a^	4.00 (3.00, 8.00)	4.00 (3.00, 8.00)	4.00 (3.00, 8.00)	−0.333	0.739
Sleep disturbance ^b^					
No	66 (51.6)	31 (48.4)	35 (54.7)	0.500	0.479
Yes	62 (48.4)	33 (51.6)	29 (45.3)		
Physical activity level ^a^					
Low	43 (33.6)	22 (34.4)	21 (32.8)	−0.292	0.770
Moderate	80 (62.5)	40 (62.5)	40 (62.5)		
High	5 (3.9)	2 (3.1)	3 (4.7)		
Drinking status ^c^					
Never	120 (93.8)	58 (90.6)	62 (96.9)	1.200	0.273
Former/current	8 (6.2)	6 (9.4)	2 (3.1)		

Data are shown as n (%), mean ± SD or median (25th and 75th percentiles). ^a^ Mann–Whitney U test. ^b^ Chi-squared test. ^c^ Chi-squared test with continuity correction. ^d^ Independent samples *t*-test. BMI, body mass index; CNY, Chinese yuan; VAS, visual analogue scale; HADS, Hospital Anxiety and Depression Scale.

**Table 3:** Linear mixed model analysis of the effects of the intervention on CRF.

Variable	Model 1		Model 2		Model 3		
	β (95% CI)	*p*	β (95% CI)	*p*	β (95% CI)	*p*	Cohen’s d
RPFS score							
Group	−0.186 (−0.780~0.408)	0.537	−0.189 (−0.782~0.404)	0.529	−0.170 (−0.750~0.410)	0.563	-
Time	−0.016 (−0.354~0.321)	0.923	−0.032 (−0.368~0.303)	0.847	−0.031 (−0.362~0.305)	0.865	-
Group × Time	−1.424 (−1.959~−0.888)	<0.001	−1.402 (−1.937~−0.867)	<0.001	−1.426 (−1.959~−0.893)	<0.001	−0.97
Behavioral fatigue							
Group	−0.444 (−1.075~0.187)	0.166	−0.441 (−1.069~0.188)	0.167	−0.421 (−1.046~0.204)	0.185	-
Time	0.008 (−0.462~0.478)	0.972	−0.019 (−0.485~0.448)	0.936	−0.023 (−0.487~0.441)	0.923	-
Group × Time	−1.240 (−1.956~−0.523)	<0.001	−1.207 (−1.922~−0.493)	0.001	−1.224 (−1.937~−0.512)	<0.001	−0.76
Affective fatigue							
Group	0.256 (−0.406~0.918)	0.445	0.248 (−0.409~0.906)	0.456	0.253 (−0.410~0.915)	0.451	-
Time	0.010 (−0.486~0.506)	0.967	0.000 (−0.496~0.496)	1.000	−0.010 (−0.503~0.482)	0.967	-
Group × Time	−1.584 (−2.277~−0.892)	<0.001	−1.561 (−2.255~−0.867)	<0.001	−1.568 (−2.259~−0.877)	<0.001	−0.92
Sensory fatigue							
Group	−0.475 (−1.154~0.201)	0.169	−0.481 (−1.159~0.197)	0.163	−0.447 (−1.099~0.205)	0.177	-
Time	0.006 (−0.448~0.461)	0.028	−0.014 (−0.467~0.438)	0.949	−0.029 (−0.477~0.420)	0.898	-
Group × Time	−1.406 (−2.054~−0.758)	<0.001	−1.378 (−2.026~−0.731)	<0.001	−1.389 (−2.032~−0.745)	<0.001	−0.79
Cognitive fatigue							
Group	−0.105 (−0.821~0.610)	0.771	−0.107 (−0.824~0.611)	0.769	−0.118 (−0.823~0.587)	0.741	-
Time	0.172 (−0.319~0.662)	0.485	0.168 (−0.323~0.658)	0.495	0.160 (−0.329~0.650)	0.513	-
Group × Time	−1.620 (−2.381~−0.859)	<0.001	−1.610 (−2.373~−0.848)	<0.001	−1.611 (−2.372~−0.851)	<0.001	−0.93

Model 1: unadjusted. Model 2: adjusted for age, BMI, and cancer stage. Model 3: additionally adjusted for pain, anxiety, and depression. RPFS, the Chinese version of the Revised Piper Fatigue Scale.

**Table 4:** Linear mixed model analysis of the effects of the intervention on CHEI.

Variable	Model 1		Model 2		Model 3		
	β (95% CI)	*p*	β (95% CI)	*p*	β (95% CI)	*p*	Cohen’s d
Group	−0.494 (−3.638~2.649)	0.756	−0.505 (−3.681~2.670)	0.753	−0.456 (−3.736~2.822)	0.783	-
Time	−0.571 (−2.824~1.681)	0.613	−0.596 (−2.845~1.653)	0.598	−0.569 (−2.817~1.679)	0.614	-
Group × Time	4.780 (1.366~8.193)	0.006	4.820 (1.408~8.235)	0.006	4.799 (1.383~8.215)	0.006	0.75

Model 1: unadjusted. Model 2: adjusted for age, BMI, and cancer stage. Model 3: additionally adjusted for pain, anxiety, and depression. CHEI, Chinese Healthy Eating Index.

**Table 5:** Linear mixed model analysis of the effects of the intervention on BMI, NRS 2002 score, and self-management efficacy.

Variable	Model 1		Model 2		Model 3		
	β (95% CI)	*p*	β (95% CI)	*p*	β (95% CI)	*p*	Cohen’s d
BMI ^a^							
Group	0.104 (−1.045~1.252)	0.858	0.128 (−1.014~1.270)	0.825	0.032 (−1.144~1.207)	0.958	-
Time	−0.025 (−0.115~0.065)	0.580	−0.024 (−0.115~0.663)	0.595	−0.023 (−0.114~0.067)	0.067	-
Group × Time	0.334 (0.106~0.562)	0.005	0.329 (0.101~0.557)	0.005	0.330 (0.102~0.558)	0.005	0.10
NRS 2002							
Group	−0.141 (−0.381~0.100)	0.250	−0.129 (−0.356~0.098)	0.263	−0.123 (−0.355~0.109)	0.297	-
Time	−0.116 (−0.276~0.043)	0.151	−0.117 (−0.276~0.042)	0.148	−0.118 (−0.277~0.041)	0.144	-
Group × Time	0.039 (−0.147~0.226)	0.677	0.035 (−0.151~0.222)	0.708	0.033 (−0.153~0.220)	0.722	0.05
SUPPH score							
Group	−1.016 (−4.396~2.365)	0.553	−1.103 (−4.486~2.281)	0.520	−0.692 (−4.124~2.740)	0.691	-
Time	4.451 (2.523~6.379)	<0.001	4.454 (2.527~6.381)	<0.001	4.457 (2.528~6.385)	<0.001	-
Group × Time	16.536 (12.435~20.638)	<0.001	16.610 (12.505~20.715)	<0.001	16.657 (12.557~20.758)	<0.001	1.65

^a^ BMI was not included as a covariate in the analysis model. Model 1: unadjusted. Model 2: adjusted for age, BMI, and cancer stage. Model 3: additionally adjusted for pain, anxiety, and depression. BMI, Body Mass Index; NRS2002, Nutrition Risk Screening 2002; SUPPH, Strategies Used by People to Promote Health.

**Table 6:** Linear mixed model analysis of the effects of the intervention on quality of life.

Variable	Model 1		Model 2		Model 3		
	β (95% CI)	*p*	β (95% CI)	*p*	β (95% CI)	*p*	Cohen’s d
FACT-B							
Group	−0.047 (−6.173~6.080)	0.998	−0.057 (−6.191~6.076)	0.985	0.424 (−4.856~5.704)	0.874	
Time	9.149 (6.898~11.401)	<0.001	9.126 (6.874~11.377)	<0.001	8.967 (6.716~11.219)	<0.001	
Group × Time	12.506 (9.074~15.938)	<0.001	12.543 (9.110~15.976)	<0.001	12.688 (9.250~16.125)	<0.001	1.16
Physical well-being							
Group	−0.813 (−2.433~0.809)	0.323	−0.815 (−2.418~0.789)	0.317	−0.691 (−2.205~0.822)	0.368	-
Time	1.449 (0.439~2.460)	0.060	1.446 (0.437~2.454)	0.006	1.476 (0.470~2.481)	0.005	-
Group × Time	1.112 (−0.530~2.754)	0.182	1.110 (−0.527~2.748)	0.182	1.121 (−0.511~2.752)	0.176	0.27
Social/family well-being							
Group	1.453 (−0.582~3.489)	0.160	1.490 (−0.560~3.541)	0.153	1.493 (−0.552~3.547)	0.151	-
Time	1.776 (0.668~2.883)	0.002	1.766 (0.658~2.874)	0.002	1.748 (0.643~2.853)	0.002	-
Group × Time	2.251 (0.498~4.003)	0.012	2.234 (0.479~3.989)	0.013	2.310 (0.558~4.061)	0.010	0.42
Emotional well-being							
Group	−0.984 (−2.766~0.797)	0.276	−0.973 (−2.762~0.815)	0.284	−0.549 (−1.989~0.891)	0.452	-
Time	0.353 (−0.603~1.309)	0.463	0.343 (−0.613~1.299)	0.475	0.271 (−0.694~1.235)	0.576	-
Group × Time	4.599 (3.224~5.975)	<0.001	4.609 (3.230~5.987)	<0.001	4.648 (3.264~6.031)	<0.001	1.16
Functional well-being							
Group	0.172 (−1.312~1.656)	0.819	0.155 (−1.327~1.638)	0.836	0.135 (−1.321~1.590)	0.855	-
Time	2.623 (1.542~3.704)	<0.001	2.615 (1.533~3.696)	<0.001	2.600 (1.523~3.678)	<0.001	-
Group × Time	2.026 (0.435~3.617)	0.013	2.050 (0.460~3.640)	0.012	2.055 (0.480~3.629)	0.110	0.52
Additional concerns							
Group	0.125 (−1.703~1.953)	0.893	0.098 (−1.740~1.937)	0.916	0.174 (−1.576~1.925)	0.844	-
Time	2.389 (1.579~3.199)	<0.001	2.372 (1.561~3.183)	<0.001	2.353 (1.541~3.164)	<0.001	-
Group × Time	2.455 (0.801~4.108)	0.004	2.508 (0.860~4.157)	0.003	2.580 (0.934~4.226)	0.002	0.59

Model 1: unadjusted. Model 2: adjusted for age, BMI, and cancer stage. Model 3: additionally adjusted for pain, anxiety, and depression. FACT-B, Functional Assessment of Cancer Therapy-Breast scale.

## Data Availability

Data are available upon reasonable request from the corresponding author. The data contain information from human participants, and access is restricted to protect participant privacy and comply with ethical regulations.

## Supplementary Materials

The supporting information can be downloaded at https://www.mdpi.com/article/10.3390/nu18060894/s1, Table S1. Patient Adherence to Online Intervention Tasks in the Intervention Group. Table S2. Changes in cancer-related fatigue between the intervention and control groups. Table S3. Changes in CHEI between the intervention and control groups. Table S4. Comparison of BMI, NRS 2002 score, and self-management efficacy between the intervention and control groups. Table S5. Changes in quality of life between the intervention and control groups.

## Abbreviations

The following abbreviations are used in this manuscript:

BMI	Body Mass Index
CHEI	Chinese Healthy Eating Index
CNY	Chinese Yuan
CONSORT	Consolidated Standards of Reporting Trials
CRF	Cancer-Related Fatigue
FACT-B	Functional Assessment of Cancer Therapy-Breast
ICD-10	International Statistical Classification of Diseases and Related Health Problems, 10th Revision
IPAQ-SF	International Physical Activity Questionnaire Short Form
IQR	Interquartile Ranges
ITHBC	Integrated Theory of Health Behavior Change
MET	Metabolic Equivalent of Task
NRS2002	Nutrition Risk Screening 2002
QoL	Quality of Life
RCT	Randomized Controlled Trial
RPFS	Revised Piper Fatigue Scale
SD	Standard Deviation
SUPPH	Strategies Used by People to Promote Health
VAS	Visual Analogue Scale
