# Supplementary material for: Effect of Nutrition Education Focusing on Dietary Quality on Cancer-Related Fatigue in Breast Cancer Patients: A 12-Week Randomized Controlled Trial
Source: Nutrients. 2026 Mar 12;18(6):894. doi: 10.3390/nu18060894 (PMC13028898; doi:10.3390/nu18060894)
Supplement: Supplementary file 1 [file nutrients-18-00894-s001.zip › nutrients-4148225-supplementary.pdf]

Table S1. *Patient Adherence to Online Intervention Tasks in the Intervention Group*

Stage	High adherence n (%)	Moderate adherence n (%)	Low adherence n (%)	Non adherence n (%)	Response rate (%)
Weeks 1–3	54 (84.4)	8 (12.5)	2 (3.1)	0 (0.0)	100.0
Weeks 4–6	49 (76.6)	9 (14.1)	3 (4.7)	3 (4.6)	95.3
Weeks 7–9	47 (77.0)	10 (16.4)	3 (4.9)	1 (1.7)	93.8
Weeks 10–12	42 (70.0)	9 (15.0)	5 (8.3)	4 (6.7)	87.5

Note: Adherence levels were classified based on the total score within each stage (maximum 16 points per stage). High adherence was defined as a total score of 13–16 points, moderate adherence as 8–12 points, low adherence as 1–7 points, and non-adherence as 0 points.

Table S2. *Changes in cancer-related fatigue between the intervention and control groups.*

Variable	Intervention (n = 56)	Control (n = 55)	*t/Z*	*p*
RPFS score				
Baseline	5.60 ± 1.71	5.85 ± 1.15	−0.970	0.332 [a]
Post-intervention	3.97 ± 1.46	5.66 ± 1.27	−5.831	<0.001 [a]
Δ RPFS	−1.63 ± 1.57	−0.19 ± 1.20	−5.477	<0.001 [a]
Z	−5.278	−1.060		
p	<0.001 [c]	0.289 [c]		
Behavioral fatigue				
Baseline	6.01 ± 1.96	6.53 ± 1.26	1.662	0.100 [b]
Post-intervention	4.56 ± 1.67	6.31 ± 1.59	5.624	<0.001 [b]
Δ Behavioral fatigue	−1.44 ± 2.09	−0.22 ± 1.69	3.398	<0.001 [b]
t	5.167	0.950		
p	<0.001 [d]	0.346 [d]		
Affective fatigue				
Baseline	5.68 ± 1.92	5.43 ± 1.53	−0.746	0.457 [b]
Post-intervention	3.91 ± 1.65	5.22 ± 1.61	−4.060	<0.001 [a]
Δ Affective fatigue	−1.77 ± 1.85	−0.21 ± 1.81	4.491	<0.001 [b]
t/Z	−5.281	0.863		
p	<0.001 [c]	0.392 [d]		
Sensory fatigue				
Baseline	5.72 ± 1.90	6.33 ± 1.46	−1.767	0.077 [a]
Post-intervention	4.15 ± 1.73	6.09 ± 1.58	−5.423	<0.001 [a]
Δ Sensory fatigue	−1.57 ± 1.77	−0.24 ± 1.61	−4.389	<0.001 [a]
Z	−4.942	−1.352		
p	<0.001 [c]	0.176 [c]		
Cognitive fatigue				
Baseline	5.08 ± 2.00	5.22 ± 1.56	0.417	0.678 [b]
Post-intervention	3.35 ± 1.75	5.11 ± 1.49	−5.293	<0.001 [a]
Δ Cognitive fatigue	−1.72 ± 2.19	−0.11 ± 1.74	4.303	<0.001 [b]
t/Z	−4.664	0.467		
p	<0.001 [c]	0.642 [d]		

Data are shown as mean ± SD. [a] Mann-Whitney U test. [b] Independent-samples t-test. [c] Wilcoxon signed-rank test. [d] Paired t-test. RPFS, the Chinese version of the Revised Piper Fatigue Scale. The change (Δ) was defined as the post-intervention score minus the baseline score for each participant.

Table S3. *Changes in CHEI between the intervention and control groups.*

Variable	Intervention (n = 56)	Control (n = 55)	*t*/*Z*	*p*
CHEI score				
Baseline	58.82 ± 9.37	60.87 ± 8.79	1.193	0.235 [a]
Post-intervention	63.42 ± 8.79	59.91 ± 9.20	−2.055	0.042 [a]
Δ CHEI	4.61 ± 10.07	−0.96 ± 8.45	−2.607	0.009 [b]
t	−3.424	0.844		
p	0.001	0.402		

Data are shown as mean ± SD. [a] Independent-samples t-test. [b] Mann-Whitney U test. [c] Paired t-test. CHEI, Chinese Healthy Eating Index. The change (Δ) was defined as the post-intervention score minus the baseline score for each participant.

Table S4. *Comparison of BMI, NRS 2002 score, and self-management efficacy between the intervention and control groups.*

Variable	Intervention (n = 56)	Control (n = 55)	*t/Z*	*p*
BMI				
Baseline	23.78 ± 3.27	23.60 ± 3.61	−0.802	0.422 [a]
Post-intervention	24.10 ± 3.00	23.58 ± 3.52	−1.383	0.167 [a]
Δ BMI	0.32 ± 0.80	−0.02 ± 0.34	−3.200	0.001 [a]
t/Z	−2.967	−0.769		
p	0.004 [d]	0.442 [c]		
NRS 2002				
Baseline	1.25 ± 0.67	1.42 ± 0.79	−1.421	0.155 [a]
Post-intervention	1.16 ± 0.50	1.27 ± 0.62	−1.112	0.266 [a]
Δ NRS 2002	−0.09 ± 0.39	−0.15 ± 0.62	−0.701	0.483 [a]
Z	−1.633	−1.582		
p	0.102 [c]	0.114 [c]		
SUPPH score				
Baseline	73.41 ± 10.91	74.36 ± 8.85	−0.390	0.697 [a]
Post-intervention	94.34 ± 13.71	78.82 ± 11.82	−6.383	<0.001 [b]
Δ SUPPH	20.93 ± 13.87	4.45 ± 7.14	−6.855	<0.001 [a]
Z	−6.095	−4.125		
p	<0.001 [c]	<0.001 [c]		

Data are shown as mean ± SD. [a] Mann−Whitney U test. [b] Independent-samples t-test. [c] Wilcoxon signed-rank test. [d] Paired t-test. BMI, Body Mass Index; NRS 2002, Nutrition Risk Screening 2002; SUPPH, Strategies Used by People to Promote Health (SUPPH) scale. The change (Δ) was defined as the post-intervention value minus the baseline value for each participant.

Table S5. *Changes in quality of life between the intervention and control groups.*

Variable	Intervention (n = 56)	Control (n = 55)	*t*/*Z*	*p*
FACT-B				
Baseline	86.11 ± 15.67	85.96 ± 16.55	−0.047	0.963 [b]
Post-intervention	108.34 ± 14.40	95.09 ± 18.67	−4.191	<0.001 [b]
Δ FACT-B	22.23 ± 9.80	9.13 ± 8.33	−7.586	<0.001 [b]
t	−16.980	−8.127		
p	<0.001 [d]	<0.001 [d]		
Physical well-being				
Baseline	17.23 ± 5.05	17.98 ± 4.01	−0.672	0.502 [a]
Post-intervention	19.80 ± 4.72	19.45 ± 4.57	−0.462	0.644 [a]
Δ Physical well-being	2.57 ± 5.02	1.47 ± 3.75	−1.548	0.122 [a]
Z	−3.948	−2.824		
p	<0.001 [c]	0.005 [c]		
Social/family well-being				
Baseline	17.98 ± 5.60	16.15 ± 5.40	−1.697	0.093 [b]
Post-intervention	22.27 ± 4.80	18.11 ± 5.74	−3.787	<0.001 [a]
Δ Social/family well-being	4.29 ± 5.30	1.96 ± 4.12	−2.577	0.011 [b]
t/*Z*	−4.849	−3.544		
p	< 0.001 [c]	<0.001 [d]		
Emotional well-being				
Baseline	13.50 ± 4.79	14.47 ± 5.20	1.026	0.307 [b]
Post-intervention	18.70 ± 3.89	15.00 ± 4.64	−4.321	<0.001 [a]
Δ Emotional well-being	5.20 ± 3.82	0.53 ± 3.60	−6.629	<0.001 [b]
t/*Z*	−6.089	−1.086		
p	<0.001 [c]	0.282 [d]		
Functional well-being				
Baseline	13.39 ± 3.97	13.65 ± 3.82	0.354	0.724 [b]
Post-intervention	18.39 ± 4.48	16.35 ± 4.90	−2.299	0.023 [b]
Δ Functional well-being	5.00 ± 4.41	2.69 ± 4.00	−2.888	0.005 [b]
t	−8.491	−4.984		
p	< 0.001 [d]	<0.001 [d]		
Additional concerns				
Baseline	24.00 ± 5.45	23.71 ± 4.33	−0.715	0.474 [a]
Post-intervention	29.18 ± 3.40	26.18 ± 4.71	−3.835	<0.001 [b]
Δ Additional concerns	5.18 ± 5.55	2.47 ± 2.99	−3.203	0.002 [b]
t/*Z*	−5.245	−6.127		
p	< 0.001 [c]	< 0.001 [d]		

Data are shown as mean ± SD. [a] Mann–Whitney U test. [b] Independent-samples t-test. [c] Wilcoxon signed-rank test. [d] Paired t-test. FACT-B, Functional Assessment of Cancer Therapy-Breast scale; The change (Δ) was defined as the post-intervention value minus the baseline value for the same individual.
